# Quantification of cardiac pumping mechanics in rats by using the elastance–resistance model based solely on the measured left ventricular pressure and cardiac output

**DOI:** 10.1007/s00424-019-02270-7

**Published:** 2019-03-23

**Authors:** Chih-Hsien Wang, Ru-Wen Chang, En-Ting Wu, Chun-Yi Chang, Hsien-Li Kao, Ming-Shiou Wu, Ya-Jung Cheng, Yih-Sharng Chen, Kuo-Chu Chang

**Affiliations:** 1 Department of Surgery, National Taiwan University Hospital, No. 7, Chung-Shan S. Rd., Taipei, 100 Taiwan; 2 Department of Physiology, College of Medicine, National Taiwan University, No. 1, Sec. 1, Jen-Ai Road, Taipei, 100 Taiwan; 3 Department of Pediatrics, National Taiwan University Hospital, No. 8, Chung-Shan S. Rd., Taipei, 100 Taiwan; 4 Department of Emergency Medicine, Taipei Veterans General Hospital, Chu-Tung Branch, Hsin-Chu, 310 Taiwan; 5 Department of Internal Medicine, National Taiwan University Hospital, No. 7, Chung-Shan S. Rd., Taipei, 100 Taiwan; 6 Department of Anesthesiology, National Taiwan University Hospital, No. 7, Chung-Shan S. Rd., Taipei, 100 Taiwan

**Keywords:** Cardiac performance, Isovolumic contraction, Triangular aortic flow, Ventricular elastance, Ventricular resistance

## Abstract

The cardiac pumping mechanics can be characterized by both the maximal systolic elastance (*E*_max_) and theoretical maximum flow (*Q*_max_), which are generated using an elastance–resistance model. The signals required to fit the elastance–resistance model are the simultaneously recorded left ventricular (LV) pressure and aortic flow (*Q*^m^), followed by the isovolumic LV pressure. In this study, we evaluated a single-beat estimation technique for determining the *E*_max_ and *Q*_max_ by using the elastance–resistance model based solely on the measured LV pressure and cardiac output. The isovolumic LV pressure was estimated from the measured LV pressure by using a non-linear least-squares approximation technique. The measured *Q*^m^ was approximated by an unknown triangular flow (*Q*^tri^), which was generated by using a fourth-order derivative of the LV pressure. The *Q*^tri^ scale was calibrated using the cardiac output. Values of *E*_max_^triQ^ and *Q*_max_^triQ^ obtained using *Q*^tri^ were compared with those of *E*_max_^mQ^ and *Q*_max_^mQ^ obtained from the measured *Q*^m^. Healthy rats and rats with chronic kidney disease or diabetes mellitus were examined. We found that the LV *E*_max_ and *Q*_max_ can be approximately calculated using the assumed *Q*^tri^, and they strongly correlated with the corresponding values derived from *Q*^m^ (*P* < 0.0001; *n* = 78): *E*_max_^triQ^ = 51.9133 + 0.8992 × *E*_max_^mQ^ (*r*^2^ = 0.8257; *P* < 0.0001); *Q*_max_^triQ^ = 2.4053 + 0.9767 × *Q*_max_^mQ^ (*r*^2^ = 0.7798; *P* < 0.0001). Our findings suggest that the proposed technique can be a useful tool for determining *E*_max_ and *Q*_max_ by using a single LV pressure pulse together with cardiac output.

## Introduction

An elastance–resistance model is an effective tool to quantify the systolic pumping mechanics of the heart in in situ, open-chest experiments [5, 14, 21, 22]. Parameters generated by this model to characterize the cardiac physical processes are maximal systolic elastance (*E*_max_) and theoretical maximum flow (*Q*_max_). Physically, *E*_max_ can represent the intrinsic myocardial contractility in an intact heart because (1) it reflects subtle changes in the contractile status of the left ventricle and (2) it is independent of the preload, afterload, and heart rate for a given cardiac contractile state [11, 24]. *Q*_max_ is the amount of outflow generated by the ventricle if it were to eject under zero-load condition, and it shares an inverse relationship with the resistive behavior of the left ventricle [20, 22].

To develop an elastance–resistance model for assessing the cardiac pumping properties, simultaneously recording the left ventricular (LV) pressure and aortic flow (*Q*^m^) followed by recording the isovolumic LV pressure is indispensable [5, 14, 21, 22]. The ascending aorta-occlusive method must be used to measure the isovolumic LV pressure at the end of diastole under open-chest conditions. Obviously, the technique for recording the isovolumic LV pressure is not permitted in human patients. In 1997, Chang and Kuo [7] used a high-fidelity multi-sensor catheter to simultaneously measure the LV pressure and aortic flow in anesthetized, closed-chest dogs. A curve-fitting technique, proposed by Sunagawa et al. [26], was performed to estimate the isovolumic LV pressure by using the recorded instantaneous LV pressure of an ejecting contraction. They discovered that an elastance–resistance model with the estimated isovolumic LV pressure can potentially be used to study the systolic pumping mechanics of the heart.

In 1989, Kelly et al. [12] found that second zero crossing of fourth-order derivative of aortic pressure is close to peak of flow. The approximation of the aortic flow to a triangle (*Q*^tri^) was reported and validated by Westerhof et al. [29]. In their study, the timing of the peak of the triangle was derived using a fourth-order derivative of the aortic pressure waveform. Using *Q*^tri^, they successfully separated the measured aortic pressure pulse into its forward and backward components. In 2017, Wang et al. [28] evaluated a method for determining the slope (*E*_es_) of the end-systolic pressure–volume relation (*ESPVR*) on the basis of the measured LV pressure and an assumed *Q*^tri^. In their study, *Q*^tri^ was derived using a fourth-order derivative of the LV (but not aortic) pressure to approximate its corresponding *Q*^m^.

The present study elaborated these concepts by determining the LV *E*_max_ and *Q*_max_ by using the measured LV pressure without recording the isovolumic LV pressure and aortic flow signals. A single-beat estimation technique was employed to calculate the *E*_max_ and *Q*_max_ by using an elastance–resistance model based solely on the measurement of the LV pressure and cardiac output. The technique proposed by Sunagawa et al. [26] was used to estimate the isovolumic LV pressure from the measured LV pressure. An uncalibrated *Q*^tri^ was constructed from the measured LV pressure based on the method proposed by Wang et al. [28]. The *Q*^tri^ scale was calibrated using the cardiac output. Values of *E*_max_^triQ^ and *Q*_max_^triQ^ obtained using *Q*^tri^ were compared with those of *E*_max_^mQ^ and *Q*_max_^mQ^ obtained from the measured *Q*^m^. Healthy rats (NC), rats with chronic kidney disease (CKD), and rats with type 1 or type 2 diabetes mellitus (DM) were analyzed. If the proposed method works out, the systolic elastic and resistive behaviors of the ventricular pump in human patients can be quantitated by using a minimally invasive measurement on the LV pressure together with non-invasive measurement on cardiac output.

## Methods

### Animals and catheterization

Male Wistar rats aged two months were divided into the following four groups: (1) NC (*n* = 25), (2) CKD (*n* = 14), (3) type 1 DM (*n* = 20), and (4) type 2 DM (*n* = 12). Female healthy rats (FNC; *n* = 7) were also included in the present study. According to the method reported by Floege et al. [9], CKD was induced through 5/6 subtotal nephrectomy (i.e., right nephrectomy and ligation of two branches of the left renal artery) in rats under anesthesia with sodium pentobarbital (50 mg kg^−1^; intraperitoneal). The levels of serum creatinine and blood urea nitrogen were determined using an autoanalyzer (Model 7070, Hitachi Electronics Co., Ltd., Tokyo, Japan). Type 1 DM was induced through a single-tail vein injection with 55 mg kg^−1^ streptozotocin (STZ; Sigma, St. Louis, MO, USA) in 0.1 M citrate buffer (pH 4.5; Sigma) [28]. Type 2 DM was induced by intraperitoneally administering 180 mg kg^−1^ nicotinamide (NA) (Sigma, St. Louis, MO, USA) 30 min before an intravenous injection of 50 mg kg^−1^ STZ dissolved in 0.1 M citrate buffer (pH 4.5) [15]. To confirm hyperglycemia, the blood glucose levels were measured using a SURESTEP Test Strip (Lifescan Inc., Milpitas, CA, USA) in the rats with induced DM. Changes in the systolic mechanical behavior of the ventricular pump were monitored 8 weeks after DM and CKD induction. All the rats were provided ad libitum Purina chow and water and housed under 12-h light–dark cycles. The experiments were conducted according to the Guide for the Care and Use of Laboratory Animals, and our study protocol was approved by the Animal Care and Use Committee of the National Taiwan University [28].

The cardiodynamic variables were measured in the anesthetized rats, according to previously described general surgical procedures and methods [28]. Briefly, the rats were anesthetized with sodium pentobarbital (50 mg kg^−1^; intraperitoneal), placed on a heating pad, intubated, and ventilated using a rodent respirator (Model 131, New England Medical Instruments, Medway, MA, USA). The chest was opened at the second intercostal space on the right side. An electromagnetic flow probe (100 series; internal circumference, 8 mm; Carolina Medical Electronics, King, NC, USA) was positioned around the ascending aorta to measure the pulsatile aortic flow. A high-fidelity pressure catheter (Model SPC 320; size, 2F; Millar Instruments, Houston, TX, USA) was inserted through the isolated right carotid artery into the left ventricle to measure the LV pressure. The electrocardiogram (ECG) of lead II was recorded using a Gould ECG/Biotach amplifier (Cleveland, OH, USA). Signals (5–10 beats at steady state) were selected on the basis of the following criteria: (1) recorded beats with optimal LV pressure and aortic velocity profiles; (2) beats with an RR interval less than 5% different from the average value for all recorded beats; and (3) exclusion of ectopic and post-ectopic beats. The selective beats were averaged in the time domain by using the peak R wave of the ECG as a fiducial point. A single-beat estimation technique was used to calculate the *E*_max_ and *Q*_max_ that characterize the systolic pumping mechanics of the heart [7].

### Construction of an isovolumic pressure and a triangular flow from the measured LV pressure

**Fig. 1:**
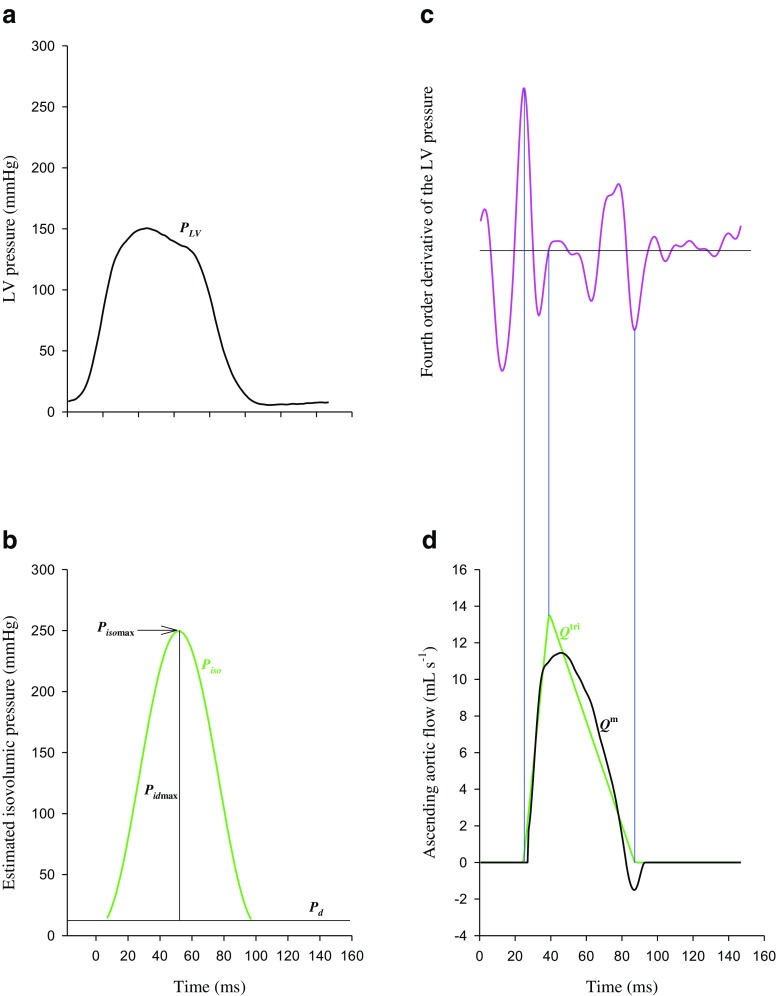
Construction of the LV isovolumic pressure (*P*_iso_, **b**) and aortic triangular flow (*Q*^tri^, **d**) from the measured LV pressure waveform (*P*_LV_, **a**) in a CKD rat. LV, left ventricular; *P*_d_, LV end-diastolic pressure; *P*_idmax_, peak developed isovolumic pressure; *P*_isomax_, peak isovolumic pressure; *Q*^m^, measured aortic flow

The isovolumic LV pressure (*P*_iso_, Fig. 1b) can be derived from the measured LV pressure of an ejection contraction (*P*_LV_, Fig. 1a) by Eq. (1) described in Appendix 1. The estimated peak isovolumic pressure (*P*_isomax_) is the pressure sum of the peak developed isovolumic pressure (*P*_idmax_) and the LV end-diastolic pressure (*P*_d_) (Fig. 1b). The uncalibrated *Q*^tri^ can be constructed by using the measured LV pressure waveform demonstrated in Appendix 2. An inverse Fourier transformation of the fourth-order derivative of the LV pressure with the first 15 harmonics (Fig. 1c) was used to determine the onset, termination, and peak time points of the triangle (green curve, Fig. 1d). The *Q*^tri^ scale was calibrated using the cardiac output.

### Calculation of the LV end-systolic elastance

**Fig. 2:**
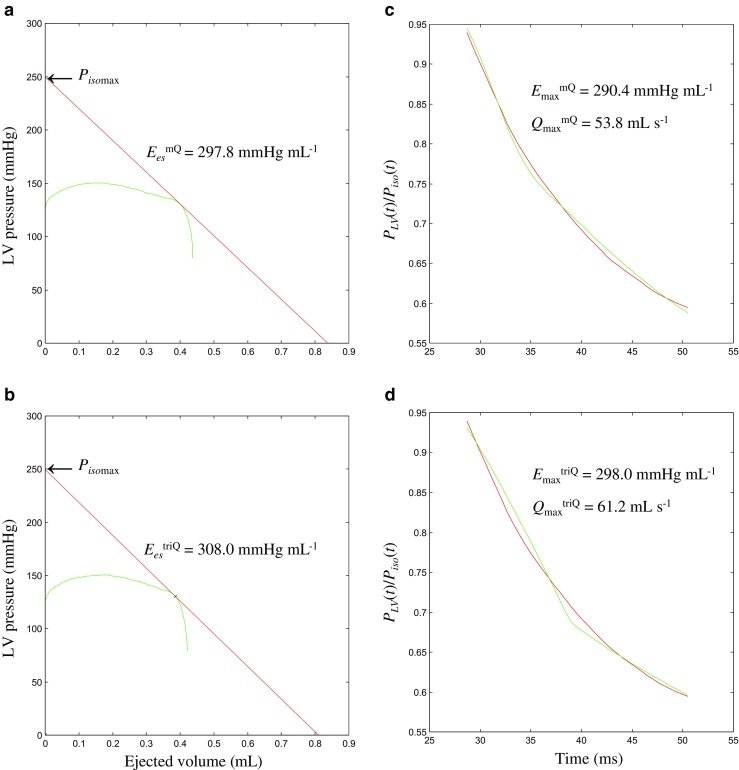
Calculation of the LV *E*_es_ and *E*_max_ from the measured *P*_LV_ in the same rat, which is shown in Fig. 1. The LV *E*_es_^mQ^ is the slope of the *ESPVsR* line, as derived from the measured *Q*^m^ (red line, **a**) and the LV *E*_es_^triQ^ from the assumed *Q*^tri^ (red line, **b**). The elastance–resistance model is used to predict the LV pressure data using either the measured *Q*^m^ (green line, **c**) or assumed *Q*^tri^ (green line, **d**), generating their corresponding LV *E*_max_^mQ^ and *E*_max_^triQ^. *E*_es_, end-systolic elastance; *E*_max_, maximal systolic elastance; *ESPVsR*, end-systolic pressure–stroke volume relationship; LV, left ventricular; *Q*_max_, theoretical maximum flow; *Q*^m^, measured aortic flow; *Q*^tri^, calibrated triangular flow; *P*_isomax_, peak isovolumic pressure; *P*_LV_, measured LV pressure

The LV end-systolic elastance (*E*_es_) can be calculated from the LV end-systolic pressure–stroke volume relationship (*ESPVsR*) that can be derived from the end-systolic pressure–volume relationship (*ESPVR*) [7, 25]. Briefly, the pressure-ejected volume loop (green curve, Fig. 2a, b) was obtained by the measured LV pressure (Fig. 1a) and the time integration of aortic flow from either *Q*^m^ (black curve, Fig. 1d) or *Q*^tri^ (green curve, Fig. 1d). In Fig. 2a, b, drawing a tangential line from the estimated *P*_isomax_ to the right corner of the pressure-ejected volume loop yields a point referred to as the end-systolic equilibrium point [2]. The line that connects the estimated *P*_isomax_ to the end-systolic equilibrium point is the *ESPVsR*, which is denoted as the red line. The slope of this red line represents the LV *E*_es_.

### Calculation of the LV maximal systolic elastance and theoretical maximum flow

The LV *E*_max_ and *Q*_max_ can be generated by using the elastance–resistance model, i.e., Eq. (2), to predict the model-derived LV pressure, which was described in Appendix 3. The signals required to fit the elastance–resistance model were the measured LV pressure, the estimated isovolumic pressure, and the aortic flow from either *Q*^m^ or *Q*^tri^. The parameters (i.e., *Q*_max_ and *V*_eed_) that coincided with the minimum objective function are considered as the model estimates of the systolic pumping mechanics of the heart (green line, Fig. 2c, d). Thus, the maximal systolic elastance of the left ventricle can be computed using the relationship *E*_max_ = *P*_isomax_ / *V*_eed_; *V*_eed_ is the effective LV end-diastolic volume. The maximal internal resistance of the left ventricle is expressed as *R*_max_ = *P*_isomax_ / *Q*_max_.

### Statistical analysis

The results are expressed as the median ± interquartile range. For comparing the effect of CKD on serum creatinine and blood urea nitrogen with that of NC, the Mann–Whitney rank-sum test was used to test for a difference between the two groups. However, the Kruskal–Wallis one-way analysis of variance (ANOVA) on ranks was performed to compare the effects of type 1 and type 2 DM on blood sugar with that of NC. The ANOVA on rank was also used to determine the statistical significance of the results for the five-group comparison on the LV pumping mechanics. Statistical significance was assumed at the level of *P* < 0.05. In cases where the ANOVA results indicated that a cardiodynamic variable differed significantly among groups, Dunn’s test was used to identify which group exhibited divergent median value from that of the NC group.

The simple linear regression is used to correlate the dependent variable (e.g., *E*_max_^triQ^) with the independent variable (e.g., *E*_max_^mQ^). The linearity of the relationship is reflected in the coefficient of determination. Larger *r*^2^ value indicates that the equation is a good description of the relation between the independent and dependent variables. The smaller *P* value denotes the greater probability that the independent variable can be used to predict the dependent variable.

Bland–Altman plots depict the difference between the two methods of measurement on the same subjects, in which good agreement is shown by values that lie close to the 0 mean difference line and between the 95% confidence interval limits of agreement [3]. The 95% limits of agreement are estimated by mean difference ± 1.96 standard deviation of the difference.

## Results

### Exemplification for constructing an isovolumic pressure and a triangular flow from the measured LV pressure

Figure 1 shows the estimated isovolumic LV pressure (b) from the measured LV pressure (a) by using a non-linear least-squares approximation technique in one male CKD rat. Figure 1 also illustrates the construction of a calibrated *Q*^tri^ (green curve, d) by using the filtered fourth-order derivative of the measured LV pressure (c).

### Exemplification for generating the end-systolic pressure–stroke volume relationship

Figure 2 depicts the calculation of the LV *E*_es_ from the measured LV pressure in the same rat, which is shown in Fig. 1. The LV *E*_es_ is the slope of the *ESPVsR* line (red line, 2a and 2b), which was obtained from the measured LV pressure (1a), the estimated isovolumic pressure (1b), and the time integration of aortic flow by using either *Q*^m^ (black curve, 1d) or *Q*^tri^ (green curve, 1d).

### Exemplification for predicting the LV pressure using the elastance–resistance model

Figure 2 also demonstrates the calculation of the LV *E*_max_ and *Q*_max_ by using the elastance–resistance model based solely on the measured *P*_*LV*_ in the same rat, which is shown in Fig. 1. The similarity between the computed (green line, 2c and 2d) and measured (red line, 2c and 2d) pressure data obtained from the measured LV pressure, the estimated isovolumic pressure, and aortic flow either from the *Q*^m^ or *Q*^tri^ is apparent. The coefficient of determination and the standard error of the estimate evaluating the goodness of the model fit using *Q*^m^ were 0.989 ± 0.005 and 2.247 ± 0.742%, respectively, and those from *Q*^tri^ were 0.987 ± 0.004 and 2.395 ± 0.443%, respectively, when all studied rats were taken into account (*n* = 78).

### LV *E*_max_ versus LV *E*_es_ in all studied rats (*n* = 78)

Although the LV *E*_es_^mQ^ (453.9 ± 117.7) was greater than the LV *E*_max_^mQ^ (445.7 ± 112.5), no statistical significance was found between these two indices describing the intrinsic contractile status of the heart. No significant difference was also observed between the LV *E*_es_^triQ^ (470.9 ± 130.7) and the LV *E*_max_^triQ^ (464.3 ± 110.8).

### Relation of the LV dynamic parameters obtained using *Q*^m^ with those generated from *Q*^tri^

**Fig. 3:**
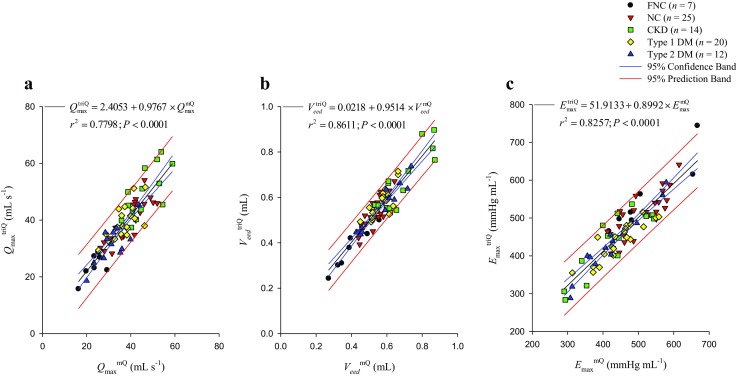
Relationship between the *Q*_max_ (**a**), *V*_eed_ (**b**), and *E*_max_ (**c**) calculated from the measured *P*_LV_ and *Q*^m^ (*Q*_max_^mQ^, *V*_eed_^mQ^, and *E*_max_^mQ^, respectively, on the horizontal axes) and the *Q*_max_, *V*_eed_, and *E*_max_ calculated from the measured *P*_LV_ and assumed *Q*^tri^ (*Q*_max_^triQ^, *V*_eed_^triQ^, and *E*_max_^triQ^, respectively, on the vertical axes). *E*_max_, maximal systolic elastance; LV, left ventricular; *P*_LV_, measured LV pressure; *Q*^m^, measured aortic flow; *Q*^tri^, calibrated triangular flow; *Q*_max_, theoretical maximum flow; *V*_eed_, effective LV end-diastolic volume; NC, normal controls; CKD, rats with chronic kidney disease; type 1 DM, streptozotocin-induced diabetic rats; type 2 DM, streptozotocin–nicotinamide-induced diabetic rats

Figure 3 displays the relationship between the *Q*_max_, *V*_eed_, and *E*_max_ calculated from the *Q*^m^ (*Q*_max_^mQ^, *V*_eed_^mQ^, and *E*_max_^mQ^, respectively, on the horizontal axes) and the *Q*_max_, *V*_eed_, and *E*_max_ calculated from the *Q*^tri^ (*Q*_max_^triQ^, *V*_eed_^triQ^, and *E*_max_^triQ^, respectively, on the vertical axes). Figure 3a displays a significant regression line for the *Q*_max_: *Q*_max_^triQ^ = 2.4053 + 0.9767 × *Q*_max_^mQ^ (*r*^2^ = 0.7798; *P* < 0.0001). Figure 3b presents the regression equation of the *V*_eed_^triQ^ = 0.0218 + 0.9514 × *V*_eed_^mQ^ (*r*^2^ = 0.8611; *P* < 0.0001). Figure 3c illustrates the regression line between the *E*_max_^triQ^ and *E*_max_^mQ^: *E*_max_^triQ^ = 51.9133 + 0.8992 × *E*_max_^mQ^ (*r*^2^ = 0.8257; *P* < 0.0001).

**Fig. 4:**
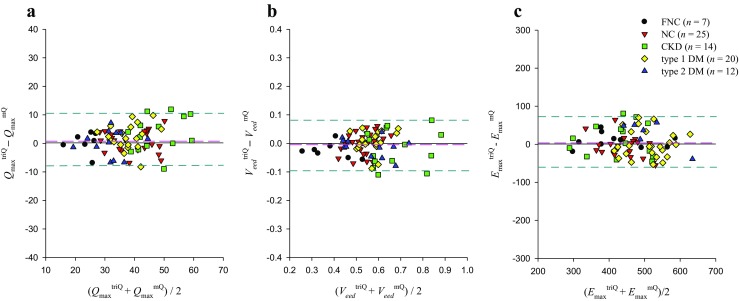
Bland–Altman plots of the *Q*_max_ (**a**), *V*_eed_ (**b**), and *E*_max_ (**c**). Dashed lines represent averages; dashed–dotted lines denote 95% confidence intervals. *E*_max_, maximal systolic elastance; LV, left ventricular; *Q*_max_, theoretical maximum flow; *V*_eed_, effective LV end-diastolic volume; NC, normal controls; CKD, rats with chronic kidney disease; type 1 DM, streptozotocin-induced diabetic rats; type 2 DM, streptozotocin–nicotinamide-induced diabetic rats

Figure 4 presents the Bland–Altman plot for the *Q*_max_ (a), *V*_eed_ (b), and *E*_max_ (c), with mean differences of 1.5441 (mL s^−1^), −0.0055 (mL), and 3.0672 (mmHg mL^−1^), respectively.

### Baseline characteristics in the studied rats

Table 1 shows the baseline characteristics of the NC, CKD, and type 1 and type 2 DM groups. Compared with the NC rats, the female healthy rats had decreased body weight associated with no change in blood sugar. The rats with CKD exhibited impaired renal function, as manifested by increased levels of serum creatinine and blood urea nitrogen. No alteration in body weight was observed in the CKD group. The rats with STZ-induced type 1 DM had higher blood glucose levels associated with a decrease in body weight compared with the NC rats. Table 1 also reveals that, partially protected by NA, the STZ-NA-induced type 2 DM elicited moderate and stable hyperglycemia and prevented STZ-induced hypoinsulinemia and body weight loss.

**Table 1:** Baseline characteristics of NC rats, rats with CKD, and rats with either type 1 or type 2 DM

Group	BW (g)	BS (mg dL^−1^)	BUN (mg dL^−1^)	SCr (mg dL^−1^)
Female				
FNC (*n* = 7)	294.7 ± 32.5^*^	104.1 ± 12.1	na	na
Male				
NC (*n* = 25)	454.5 ± 56.4	99.0 ± 11.1	20.3 ± 5.8	0.67 ± 0.13
CKD (*n* = 14)	416.1 ± 59.8	na	66.7 ± 13.4^*^	1.72 ± 0.50^*^
Type 1 DM (*n* = 20)	328.8 ± 41.1^*^	465.5 ± 44.8^*^	na	na
Type 2 DM (*n* = 12)	412.4 ± 50.8	158.4 ± 26.7^*^	na	na

All values are expressed as the median ± interquartile range. BW, body weight; BS, blood sugar; BUN, blood urea nitrogen; SCr, serum creatinine; NC, normal controls; CKD, rats with chronic kidney disease; type 1 DM, streptozotocin-induced diabetic rats; type 2 DM, streptozotocin–nicotinamide-induced diabetic rats; na, not applicable

*P < 0.05 compared with the male controls

### Basic hemodynamic measurements in the studied rats

Table 2 presents the basic hemodynamic measurements of the NC, CKD, and type 1 and type 2 DM groups. Compared with the NC group, the female healthy rats had a decline in cardiac output and peak isovolumic pressure, but no change in heart rate. No alterations in the heart rate, cardiac output, and peak isovolumic pressure were observed in the rats with CKD. The type 1 (but not type 2) DM group exhibited a significant reduction in heart rate. However, the type 2 (but not type 1) DM group showed a decline in cardiac output and peak isovolumic pressure.

**Table 2:** Basic hemodynamic data of NC rats, rats with CKD, and rats with either type 1 or type 2 DM

Group	*HR* (beats min^−1^)	*CO* (mL s^−1^)	*P*_isomax_ (mmHg)
Female			
FNC (*n* = 7)	378.5 ± 43.1	1.290 ± 0.594^*^	186.7 ± 61.4^*^
Male			
NC (*n* = 25)	398.0 ± 38.3	2.260 ± 0.619	263.0 ± 24.6
CKD (*n* = 14)	385.2 ± 51.3	2.231 ± 0.324	284.2 ± 34.8
Type 1 DM (*n* = 20)	342.7 ± 36.8^*^	2.289 ± 0.441	247.7 ± 45.8
Type 2 DM (*n* = 12)	384.5 ± 67.4	1.793 ± 0.670^*^	240.9 ± 53.8^*^

All values are expressed as the median ± interquartile range. *HR*, basal heart rate; *CO*, cardiac output; *P*_isomax_, peak isovolumic pressure; NC, normal controls; CKD, rats with chronic kidney disease; type 1 DM, streptozotocin-induced diabetic rats; type 2 DM, streptozotocin–nicotinamide-induced diabetic rats

*P < 0.05 compared with the male controls

### Effects of sex and CKD and type 1 or type 2 DM on LV pumping dynamics

**Fig. 5:**
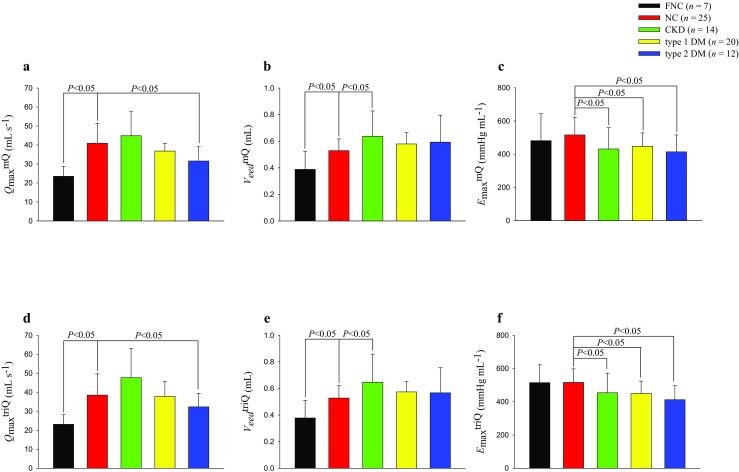
Effects of sex, CKD, and type 1 and type 2 DM on *Q*_max_^triQ^ (**d**), *V*_eed_^triQ^ (**e**), and *E*_max_^triQ^ (**f**), as derived from the assumed *Q*^tri^, showed similar statistical significance to those of their measured counterparts, i.e., *Q*_max_^mQ^ (**a**), *V*_eed_^mQ^ (**b**), and *E*_max_^mQ^ (**c**), as calculated from the measured *Q*^m^. All values are expressed as the median ± interquartile range. *E*_max_, maximal systolic elastance; LV, left ventricular; *P*_LV_, the measured LV pressure; *Q*^m^, measured aortic flow; *Q*^tri^, calibrated triangular flow; *V*_eed_, effective LV end-diastolic volume; *Q*_max_, theoretical maximum flow; FNC, female NC; NC, normal controls; CKD, rats with chronic kidney disease; type 1 DM, streptozotocin-induced diabetic rats; type 2 DM, streptozotocin–nicotinamide-induced diabetic rats. *P < 0.05 compared with the controls

Figure 5 illustrates the effects of sex and the experimental induced CKD and type 1 and type 2 DM on the systolic mechanical behavior of the ventricular pump, as derived from either the *Q*^m^ or *Q*^tri^. Compared with the NC group, the female healthy group had decreased *Q*_max_^mQ^ and *V*_eed_^mQ^ but showed no alteration in *E*_max_^mQ^. No statistical significant difference in *Q*_max_^mQ^ was found between the CKD group and the NC group. However, the CKD group showed an increase in the *V*_eed_^mQ^ and a decrease in the *E*_max_^mQ^. The type 2 (but not type 1) DM group exhibited a significantly lower *Q*_max_^mQ^ than the NC group did. Both DM groups showed no alteration in the *V*_eed_^mQ^ but showed a decline in the *E*_max_^mQ^. Meanwhile, the female healthy group, the CKD group, and both diabetic groups exhibited a difference in the *Q*_max_^triQ^, *V*_eed_^triQ^, and *E*_max_^triQ^, which showed similar statistical significance to that of their measured counterparts (i.e., *Q*_max_^mQ^, *V*_eed_^mQ^, and *E*_max_^mQ^, respectively).

## Discussion

The myocardium of the left ventricle is a viscoelastic material whose mechanical properties are reflected in the behavior of the ventricular chamber (i.e., the relationships among chamber pressure, volume, and flow). The relationship between instantaneous ventricular pressure and volume, the so-called pure elastance model, has been described in terms of a time-varying elastance [16, 24]. For an ejecting beat, the LV end-systolic elastance can be derived from the pure elastance model at the end of systole, the time at which the time-varying elastance becomes maximal [16, 23]. However, this end-systolic elastance is only an approximation to the maximal systolic elastance that is derived from isovolumic contraction, due to deactivation of myocardial shortening [6, 10, 18]. Hunter et al. [11] and Shroff et al. [17] attempted to identify the mechanical nature of the ejecting ventricle by developing models reasonably able to predict pressure–volume–flow dynamics. Campbell et al. [4] and Shroff and Motz [21] arrived at an identical configuration—a series combination of a time-varying elastance and a viscous resistance to formulate the elastance–resistance model.

### Pure elastance model versus elastance–resistance model

The LV systolic elastance can be calculated from either the pure elastance model, indicated as *E*_es_, or the elastance–resistance model, denoted as *E*_max_. However, only the elastance–resistance model has the ability to generate the LV *Q*_max_ having an inverse relationship with the LV internal resistance. At the molecular level, the LV systolic elastance can be determined by the properties of the contractile unit along with the activation process (i.e., availability of Ca^2+^) and extramyocytic components [19]. However, the ventricular resistance seems to be related to the biochemical alterations of the myocardium, having an inverse relation with percent slow myosin [19].

The pure elastance model is a model-independent approach; the elastance–resistance model is a model-based approach. The major difference between these two approaches is that the original pure elastance model can accurately predict stroke volume but not instantaneous flow; for a reasonably accurate prediction of instantaneous flow, a series resistance has to be added [21]. It should be noted that the choice of a model-independent versus model-based approach clearly depends on the specific application. If the framework of an analysis involves mean values of relevant variables (e.g., stroke volume, stroke work), the pure elastance model may be appropriate [25]. If the analysis goals are such that instantaneous behavior is of significance (e.g., coupling from the perspective of pulsatile energy generated by the left ventricle, effects of arterial wave reflection), the elastance–resistance model may be more appropriate [19].

### Evaluation of systolic pumping mechanics of the heart using a single LV pressure pulse

As mentioned earlier, the signals required to fit the elastance–resistance model are the simultaneously recorded LV pressure and aortic flow, followed by the isovolumic LV pressure. Obviously, the technique of measuring the isovolumic signal by occluding the ascending aorta limits its applicability in human patients. That may be the reason why a significance of the elastance–resistance model was not appreciated in a field a lot. In the present study, we proposed a technique to generate the essential signals for fitting the elastance–resistance model on the basis of the measured LV pressure alone. The first one estimated the isovolumic LV pressure from the LV pressure pulse of the ejecting beat. The second step generated a triangular flow from the measured LV pressure to approximate its corresponding aortic flow.

### Construction of an isovolumic pressure and a triangular flow from the measured LV pressure

In 1980, Sunagawa et al. [26] proposed a curve-fitting technique to estimate the isovolumic LV pressure from the instantaneous pressure of an ejecting contraction (Appendix 1). They discovered that the isovolumic LV pressure and its peak value could be estimated with reasonable accuracy within the interval *t*_ej_ < *t* < *t*_pisomax_, where *t*_ej_ is the onset of ventricular ejection and *t*_pisomax_ is the time of peak isovolumic pressure.

A triangular shape of the aortic flow wave is a reasonable assumption because the triangular approximation is consistent with the general aortic flow envelope obtained from Doppler ultrasound [30] or electromagnetic [29] flow measurements. In 2006, Westerhof et al. [29] provided a novel method for separating the measured aortic pressure wave into its forward and backward components on the basis of a single aortic pressure pulse and an assumed triangular flow. The attractiveness of their study is that calibration of the triangular flow wave derived from the measured aortic pressure is not essential in the analysis. In 2017, Wang et al. [28] proposed a method to construct an aortic triangular flow by using the measured LV pressure (Appendix 2). To investigate the cardiac contractile mechanics, the assumed triangular flow has to be calibrated with the cardiac output. In the present study, the LV pressure pulse was the only signal measured. Thus, the method proposed by Wang et al. [28] was employed to generate the *Q*^tri^ for determining the systolic elastic and resistive behaviors of the heart. The similarity between the aortic flow pattern of the assumed *Q*^tri^ and that of the measured *Q*^m^ was demonstrated in Fig. 1d.

### Restriction of the LV pressure data on the fitting interval *t*_ej_ < *t* < *t*_pisomax_

Hunter et al. [11] have demonstrated three components in the LV response to a flow pulse; elastance, resistance, and deactivation. Without considering the deactivation factor, our model-based approach was highly dependent on the ventricular elastance and resistance. Although not perfect, the elastance–resistance model can be used to fit the measured LV pressure of an ejecting beat suitably if the fitting interval is *t*_ej_ < *t* < *t*_pisomax_ (Appendix 3) [5]. That is because the deactivation component was virtually absent during early systole, became evident during mid-systole, and was most pronounced during late systole [11]. In the present study, we demonstrated that the elastance–resistance model with either the *Q*^m^ or *Q*^tri^ could be satisfactorily applied to measure the intrinsic LV systolic mechanics within the specified fitting interval. The LV *E*_max_ and *Q*_max_ calculated using the assumed *Q*^tri^ strongly correlated with the corresponding values derived from the measured *Q*^m^ (*Q*_max_^triQ^ vs. *Q*_max_^mQ^, Fig. 3a; *E*_max_^triQ^ vs. *E*_max_^mQ^, Fig. 3c).

### Physiological meaning of the model-generated parameters for CKD and type 1 or type 2 DM

As mentioned earlier, the LV *E*_max_ was determined by the ratio of peak isovolumic pressure to the effective end-diastolic volume. Compared with the NC group, the female healthy group had diminished effective end-diastolic volume (*V*_eed_^mQ^, Fig. 5b; *V*_eed_^triQ^, Fig. 5e) with a reduction in the peak isovolumic pressure (Table 2), resulting in no alteration in the *E*_max_ (*E*_max_^mQ^, Fig. 5c; *E*_max_^triQ^, Fig. 5f). By contrast, a decline in *Q*_max_ (*Q*_max_^mQ^, Fig. 5a; *Q*_max_^triQ^, Fig. 5d) was observed in the female healthy rats.

In the CKD group, the increased effective end-diastolic volume (*V*_eed_^mQ^ or *V*_eed_^triQ^) without any significant change in the peak isovolumic pressure contributed to a reduction in the *E*_max_ (*E*_max_^mQ^ or *E*_max_^triQ^). These results indicate that the myocardium is incapable of producing the pressure force enough to support *E*_max_ along with the increased effective end-diastolic volume. Thus, the CKD heart can be characterized as a weaker pressure generator. Although no statistical significance was found, the CKD group exhibited an increase in the *Q*_max_ (*Q*_max_^mQ^ or *Q*_max_^triQ^) compared with the corresponding respective values in the NC group. An increase in *Q*_max_ is indicative of a decrease in LV internal resistance, which can enhance ventricular outflow for a given cardiac contractile status and arterial load [18]. Therefore, the left ventricle of CKD can be characterized as a stronger flow generator. The opposing effects of reduced *E*_max_ and increased *Q*_max_ may negate each other, and, then, the cardiac pumping function of the CKD rats could be preserved before heart failure occurs.

In both diabetic groups, there was a trend toward decreasing peak isovolumic pressure (especially the type 2 DM) and increasing effective end-diastolic volume (*V*_eed_^mQ^ or *V*_eed_^triQ^), leading to a reduction in the *E*_max_ (*E*_max_^mQ^ or *E*_max_^triQ^). These results indicate that the myocardium cannot generate the pressure force enough to support *E*_max_ along with the increased effective end-diastolic volume. Thus, the diabetic heart can be characterized as a weaker pressure generator. A decline in *Q*_max_ (*Q*_max_^mQ^ or *Q*_max_^triQ^) was also observed in both diabetic groups (especially the type 2 DM). A decrease in *Q*_max_ is indicative of an increase in LV internal resistance, which can reduce ventricular outflow for a given cardiac contractile state and arterial load [18]. Therefore, the diabetic heart can be characterized as a weaker flow generator. The decreased *E*_max_ and *Q*_max_ demonstrate a deterioration in systolic pumping function of the diabetic heart. Changes in the *E*_max_ and *Q*_max_, assessed using either the *Q*^m^ or *Q*^tri^, suggested that CKD and DM could modify the systolic elastic and resistive behaviors of the left ventricle.

### Limitations

Hunter et al. [11] have demonstrated that besides elastance and resistance, there are at least two or more processes involved in the description of systolic mechanical behavior of the ventricular pump. These processes may include the volume influence factor and the deactivation factor. Campbell et al. [4, 5] provided clear evidence of failings of the elastance–resistance model, especially during late systole. However, they demonstrated that the elastance–resistance model could be satisfactorily used to fit the measured LV pressure of an ejecting beat in the fitting interval *t*_ej_ < *t* < *t*_pisomax_. Although not perfect, the elastance–resistance model can provide such a succinct representation of the systolic mechanical behavior of the ventricular pump. Furthermore, Shroff et al. [20] reported that the elastance–resistance model is a useful tool to quantify the systolic pumping mechanics of the left ventricle, provided the limitations of this model are clearly understood.

## Conclusions

The LV *E*_max_ and *Q*_max_ can be determined by using a minimally invasive measurement on the LV pressure. The aortic flow and isovolumic LV pressure necessary to fit the elastance–resistance model are both derived from the measured LV pressure together with cardiac output. We discovered that the estimated triangular flow can approximately calculate the LV *E*_max_ and *Q*_max_ in healthy rats and rats with CKD and type 1 or type 2 DM, and they had a strong correlation with the corresponding values derived from the measured aortic flow. The left ventricle of CKD can be characterized as a weaker pressure generator (decreased *E*_max_) but stronger flow generator (increased *Q*_max_). By contrast, the diabetic heart can be characterized as a weaker pressure (decreased *E*_max_) and flow (decreased *Q*_max_) generator. We suggest that the single-beat estimation technique is an effective method for calculating the *E*_max_ and *Q*_max_ by using a single LV pressure pulse recording together with cardiac output.

### Perspectives

Although the LV pressure and aortic flow can be simultaneously measured using a high-fidelity micromanometer (such as model SVPC-664D, Millar Instruments Inc., USA) in human patients, the catheter and its associated instruments are expensive and not widely available. Moreover, the flow signal in the ascending aorta is not easy to access because of the small magnitude with noise disturbances. Although the aortic flow can non-invasively be obtained by Doppler ultrasound [30], there is difficulty in “simultaneously recording the LV pressure and aortic flow” for fitting the elastance–resistance model. In the present study, both the aortic flow and isovolumic LV pressure were derived from the only measured LV pressure together with cardiac output. In clinical settings, it is easier and safer to measure cardiac output by using non-invasive impedance cardiography [1] or echocardiography [13] to calibrate the triangular flow scale. The novelty of such an approach is that one can compute the ventricular elastance and resistance without any measurements of the ascending aortic flow wave and LV pressure from isovolumic contraction. The single-beat estimation technique can be used to investigate the cardiac pumping mechanics from solely the LV pressure of an ejection contraction obtained over a single cardiac cycle without any perturbations of the loading conditions. Thus, the practical applicability of this study is that one may evaluate the LV systolic mechanical properties in patients by using a single LV pressure measurement together with cardiac output, because the generation of the isovolumic LV pressure and *Q*^tri^ and the calculation of the LV *E*_max_ and *Q*_max_ can be automatically achieved.

## Appendix 1

### Estimation of the isovolumic pressure from an ejecting contraction

The isovolumic LV pressure (*P*_iso_, Fig. 1b) was derived from the measured LV pressure of an ejection contraction (*P*_LV_, Fig. 1a) by using a non-linear least-squares approximation technique as follows [26]:

1 $$ {P}_{\mathrm{iso}}(t)=\frac{1}{2}{P}_{\mathrm{id}\max}\left[1-\cos \left(\omega t+c\right)\right]+{P}_{\mathrm{d}} $$

where *P*_idmax_ is the peak developed isovolumic pressure, *ω* is the angular frequency, *c* is the phase-shift angle of the sinusoidal curve, and *P*_d_ is the LV end-diastolic pressure. The *P*_iso_(*t*) was obtained by fitting the measured *P*_LV_ segments from the end-diastolic pressure point to the peak +*dP*_LV_/*dt* and from the pressure point of the peak −*dP*_LV_/*dt* to the same level as the end-diastolic pressure of the preceding beat [27]. The peak of the ECG R wave was used to identify the LV end-diastolic point. The estimated peak isovolumic pressure, *P*_isomax_, is the pressure sum of the peak developed isovolumic pressure and the LV end-diastolic pressure (Fig. 1b).

## Appendix 2

### Construction of the unknown flow wave by using a triangle

The fourth-order derivative of the LV pressure waveform (Fig. 1c) was filtered to determine the uncalibrated *Q*^tri^ [28]. This calculation was performed using an inverse Fourier transformation of the fourth-order derivative of the LV pressure with the first 15 harmonics. The *Q*^tri^ onset was identified using the peak of the pink curve near the end of the isovolumic contraction period (first vertical blue line). The *Q*^tri^ termination was identified using the nadir of the pink curve, near the middle of the isovolumic relaxation period (third vertical blue line). The base of the unknown *Q*^tri^ was subsequently constructed using the duration identical to the time interval between the onset and termination of the *Q*^tri^. After the ejection commenced, the first zero crossing from negative to positive (second vertical blue line) determined the peak of the triangle [28]. The *Q*^tri^ scale was calibrated using the cardiac output. Thus, the unknown flow wave *Q*^tri^ was approximated to a triangle (green curve, Fig. 1d).

## Appendix 3

### Prediction of the LV pressure using the elastance–resistance model

The model-derived LV pressure ${\hat{P}}_{LV}(t)$ can be predicted using the elastance–resistance model if the model parameters are previously identified [5, 20]. The relationship among the instantaneous LV pressure (*P*_LV_), aortic flow (*Q*), and isovolumic LV pressure (*P*_iso_) can be written as follows:

2 $$ {\hat{P}}_{LV}(t)=P{}_{iso}(t)\left[1-\frac{V_{ej}(t)}{V_{eed}}\right]\left[1-\frac{Q(t)}{Q_{\mathrm{max}}}\right] $$

where *V*_ej_(*t*) is the instantaneously ejected volume computed by calculating the running integral of the *Q*(*t*) from either the *Q*^tri^ or *Q*^m^ and *V*_eed_ is the effective LV end-diastolic volume, that is, the difference between the LV end-diastolic volume and the dead volume. The dead volume is the theoretical value below which the ventricle cannot generate any supra-atmospheric pressure. *Q*_max_ is the theoretical maximum flow that could be generated under zero load condition, and not the maximum of the flow trace in the ascending aorta.

Both the *V*_eed_ and *Q*_max_ are model parameters determined by curve-fitting techniques. Campbell et al. [5] demonstrated that Eq. (2) can be used to fit the measured LV pressure of an ejecting beat suitably if the fitting interval is *t*_ej_ < *t* < *t*_pisomax_, where *t*_ej_ is the onset of ventricular ejection and *t*_pisomax_ is the time of peak isovolumic pressure. Initial values of *V*_eed_ and *Q*_max_ were chosen first. The Nelder–Mead simplex algorithm [8] was, then, used to iteratively adjust the *V*_eed_ and *Q*_max_ to minimize the root-mean-square error [7]. The parameters that coincided with the minimum objective function were considered the model estimates of the systolic pumping mechanics of the heart (Fig. 2c, d). Goodness of the model fit can be reflected in the coefficient of determination (r^*2*^) and the standard error of the estimate (*SEE*), which were calculated from a linear regression of the model-generated pressure on measured pressure values. The LV systolic elastance was computed using the relationship *E*(*t*) = *P*_iso_(*t*) / *V*_eed_, and its maximal value was the maximal systolic elastance (*E*_max_ = *P*_isomax_ / *V*_eed_). The LV internal resistance was expressed as *R* (*P*_iso_) = *P*_iso_(*t*) / *Q*_max_.

## Abbreviations

CKD: chronic kidney disease
DM: diabetes mellitus
*E*_es_: end-systolic elastance (mmHg mL^−1^)
*E*_es_^mQ^: *E*_es_ calculated from the LV pressure and *Q*^m^
*E*_es_^triQ^: *E*_es_ calculated from the LV pressure and *Q*^tri^
*E*_max_: maximal systolic elastance (mmHg mL^−1^)
*E*_max_^mQ^: *E*_max_ calculated from the LV pressure and *Q*^m^
*E*_max_^triQ^: *E*_max_ calculated from the LV pressure and *Q*^tri^
LV: left ventricular
NA: nicotinamide
*Q*^m^: measured aortic flow wave (mL s^−1^)
*Q*^tri^: assumed triangular flow wave (mL s^−1^)
*Q*_max_: theoretical maximum flow (mL s^−1^)
*Q*_max_^mQ^: *Q*_max_ calculated from the LV pressure and *Q*^m^
*Q*_max_^triQ^: *Q*_max_ calculated from the LV pressure and *Q*^tri^
STZ: streptozotocin
*V*_eed_: effective end-diastolic volume (mL)
*V*_eed_^mQ^: *V*_eed_ calculated from the LV pressure and *Q*^m^
*V*_eed_^triQ^: *V*_eed_ calculated from the LV pressure and *Q*^tri^
